# Effects of Long-Term Exposure to 2260 m Altitude on Working Memory and Resting-State Activity in the Prefrontal Cortex: A Large-Sample Cross-Sectional Study

**DOI:** 10.3390/brainsci12091148

**Published:** 2022-08-28

**Authors:** Xin Chen, Aibao Zhou, Junle Li, Bing Chen, Xin Zhou, Hailin Ma, Chunming Lu, Xuchu Weng

**Affiliations:** 1 Key Laboratory of Brain, Cognition and Education Sciences, Ministry of Education, South China Normal University, Guangzhou 510631, China; 2 School of Psychology, South China Normal University, Guangzhou 510631, China; 3 Institute for Brain Research and Rehabilitation, South China Normal University, Guangzhou 510631, China; 4 Research Center of Plateau Brain Science, Tibet University/South China University, Guangzhou 510631, China; 5 College of Psychology, Northwest Normal University, Lanzhou 730070, China; 6 Jing Hengyi School of Education, Hangzhou Normal University, Hangzhou 311121, China; 7 Center for Cognition and Brain Disorders, The Affiliated Hospital of Hangzhou Normal University, Hangzhou 311121, China; 8 Plateau Brain Science Research Center, Tibet University/South China Normal University, Lhasa 850012, China; 9 State Key Laboratory of Cognitive Neuroscience and Learning and IDG/McGovern Institute for Brain Research, Faculty of Psychology, Beijing Normal University, Beijing 100875, China

**Keywords:** moderate altitude, working memory, fNIRS, resting state, brain networks, complexity

## Abstract

It has been well established that very-high-altitude (>4000 m) environments can affect human cognitive function and brain activity. However, the effects of long-term exposure to moderate altitudes (2000–3000 m) on cognitive function and brain activity are not well understood. In the present cross-sectional study, we utilized an N-back working memory task and resting-state functional near-infrared spectroscopy to examine the effects of two years of exposure to 2260 m altitude on working memory and resting-state brain activity in 208 college students, compared with a control group at the sea level. The results showed that there was no significant change in spatial working memory performance after two years of exposure to 2260 m altitude. In contrast, the analysis of resting-state brain activity revealed changes in functional connectivity patterns in the prefrontal cortex (PFC), with the global efficiency increased and the local efficiency decreased after two years of exposure to 2260 m altitude. These results suggest that long-term exposure to moderate altitudes has no observable effect on spatial working memory performance, while significant changes in functional connectivity and brain network properties could possibly occur to compensate for the effects of mild hypoxic environments. To our knowledge, this study is the first to examine the resting state activity in the PFC associated with working memory in people exposed to moderate altitudes.

## 1. Introduction

With the globalization of economy and culture, population mobility between highlands and plains has greatly increased. As a result, over 500 million people live above an altitude of 1500 m on Earth nowadays; among them, about 95% live between 1500 m and 3000 m [1]. In addition, 2000–2500 m is considered to be the optimal altitude range for athletes to train to enhance their endurance performance [2].

High-altitude environments can affect human cognitive function even at 1500 m [3,4,5,6]. The effects of high-altitude exposure, especially at very high altitudes (>4000 m), on human cognitive function have been relatively well investigated in previous studies, with many reported executive function impairments [7,8,9]. However, very few studies have examined the effects of exposure to moderate altitudes (2000 m–3000 m) on cognitive function and brain activity, even though the majority of people living at high altitudes live in this altitude range. While some studies on acute exposure to moderate altitudes reported cognitive impairments [10,11], others found no impairments or even found cognitive benefits [12,13,14,15,16]. Several reasons appear to account for these inconsistent results. First, the effects of acute exposure to moderate altitudes on cognition may be more subtle compared with those of high altitude. Second, the variation in research methods makes it difficult to compare findings across different studies. Finally, considering individual differences and the existence of compensatory mechanisms, the small sample sizes may also contribute to the inconsistent results [17].

Notably, there are very few studies on long-term exposure. Our previous behavioral study showed that no significant changes were found in most cognitive functions (including new verbal learning and memory, working memory, long-term explicit memory, implicit procedural learning and short- and long-term visual memory) after six months of exposure to 2260 m altitude, except for visual perception [18]. In addition, a recent study using the Go/NoGo paradigm with event-related potential (ERP) showed that no changes were found in behavioral performance, while the amplitude of P3 was significantly decreased after two years of exposure to 2950 m [19]. Adaptation based on compensatory mechanisms may account for the preserved cognitive performance. In contrast, previous studies showed that the alteration of resting state networks was correlated with the impairment of executive function after two years of exposure to high altitude (3650 m) [20,21]. However, we still do not know the neural adaptive mechanisms of the resting-state brain underlying the long-term exposure to moderate altitude.

The present study focused on the prefrontal cortex (PFC) with a large sample size. This is because a large number of studies have consistently shown that long-term exposure to high altitude impairs the PFC functions [19,22,23]. The PFC plays an important role in executive function, which includes general cognitive control, task switching and working memory updating [24]. Similar to many studies, a spatial N-back task was used to investigate the effects of long-term (two years) exposure to moderate altitude (2260 m) on working memory updating, a representative function of the PFC functions. More importantly, we also investigated the resting-state functional connectivity, brain networks and complexity within the PFC. Previous studies have demonstrated that the functional connectivity and brain network in the PFC are substantially influenced by cognitive challenges and psychiatric disorders [25,26]. Specifically, we utilized functional near-infrared spectroscopy (fNIRS) to investigate the effect of long-term exposure to 2260 m altitude on spontaneous neural activity in the PFC. With its portability and operability, fNIRS has high ecological validity and can be used on a large sample. Remarkably, we used the same set of fNIRS instruments in all participants reported here. Graph theory-based resting-state functional network analysis and sample entropy-based brain signal complexity analyses were used to examine the changes in spontaneous brain activity in the PFC. Based on the above literature review, we hypothesized that two years of exposure to 2260 m altitude would not show a significant impairment of working memory performance but would induce adaptive changes in brain activity in the PFC.

## 2. Materials and Methods

### 2.1. Participants

A total of 208 third-year college students from two altitudes participated in this study. The control group (*n* = 91) was sea level residents from Hangzhou Normal University (19 m, Hangzhou, China), who always lived in regions near the sea level (<1000 m). The experimental group (*n* = 117) was high landers from Qinghai Minzu University (2260 m, Xining, China), who had migrated from the sea level (<1000 m) to Xining for university education for about two years before the experiment. None of the participants had been to the highlands before entering university. All the participants were right-handed and of Han nationality. The age and sex ratios were not statistically different between the two groups (Table 1). The participants did not have any psychiatric or neurological disorders. We calculated the sample size using G*Power software (Ver. 3.1.9.4; Franz Paul, Kiel, Germany) based on effect size, α and Power (1 − β). With the three parameters set to 0.5, 0.05 and 0.8, respectively, the calculated sample size was 128, and with the significance level α set to 0.01, the calculated sample size increased to 192. The study was conducted in accordance with the Declaration of Helsinki and was approved by the Ethics Committee of the Institute for Brain Research and Rehabilitation at South China Normal University and the Research Center of Plateau Brain Science of Tibet University/South China University. All participants provided written informed consent before the experiment.

### 2.2. Physiological and Behavior Tests

The Raven’s Progressive Matrices (RPMs) were used to measure the general cognitive functions. The heart rate (HR) and peripheral oxygen saturation (SpO_2_) were measured using a finger pulse oximeter (YX302, Yuyue Co., Ltd., Danyang China). No significant differences were found in the RPMs between the two groups. As expected, the SpO_2_ and HR were significantly decreased in the Xining group compared with those in the Hangzhou group (Table 1).

**Figure 1:**
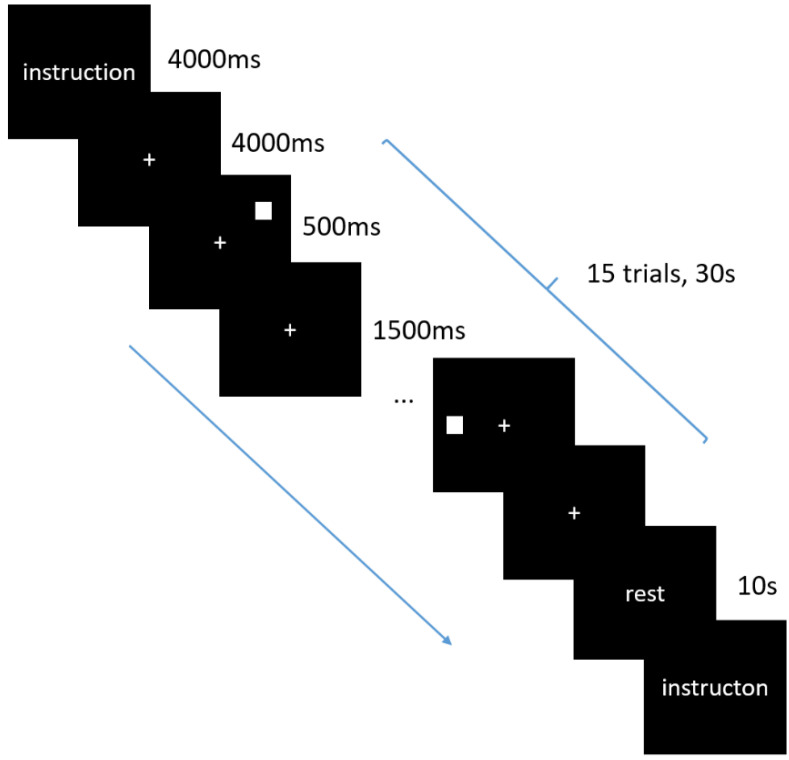
Schematic illustration of the N-back working memory task. The central crosses are the points of fixation and the white squares are the target stimulus. The arrow indicates the order of stimulus presentation.

An N-back paradigm was used to assess the working memory performance. During the spatial N-back task, a white square of one centimeter on a black background was presented on the screen [27]. In each trial, the white square was randomly presented in one of the eight positions around the central cross (up, down, left, right, upper-left, upper-right, lower-left, lower-right). The participants were required to press a button to determine whether the current stimulus position was the same as the stimulus presented for the 1 or 2 screen previously for the 1- or 2-back blocks, respectively. To control for sensorimotor effects, there was a control condition (0-back), during which the participants were required to determine whether the current position of the square was above the central across. The stimulus was presented for 500 ms, with an ISI (interstimulus interval) of 1500 ms. The task was block-designed; each condition involved 5 blocks with 15 trials. Prior to each block, there was a 4000 ms introduction screen which indicated the condition of the present block, and this was followed by a 4000 ms blank screen. The screen was approximately 70 cm from the participants’ eyes. The duration of each block was 38 s, with an interval of 10 s between blocks (Figure 1).

### 2.3. fNIRS Data Acquisition

**Figure 2:**
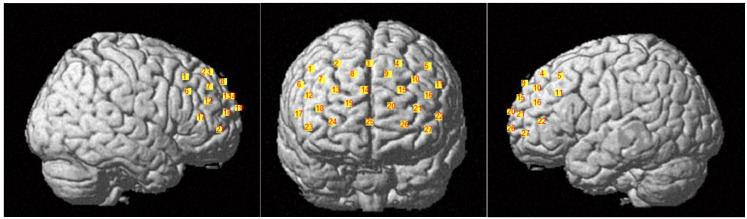
Positions of fNIRS probe set. The number on the yellow square background represents the position of the 27 channels.

The experiments were performed in quiet rooms. During the experiments, the participants were asked to keep still, relax their mind and close their eyes. The CW6 fNIRS system (Techen Inc., Milford, MA, USA) was used to record the hemodynamic concentration changes in the resting state at two laboratories (one in Hangzhou and the other in Xining). Two wavelengths (690 nm and 830 nm) were used to measure changes in oxy-haemoglobin (HbO) and deoxy-haemoglobin (HbR) concentrations in each CH, with a sampling rate of 25 Hz. A probe set of 27 channels (9 sources and 9 detectors, 3 cm distance) was placed on the prefrontal cortex of the brain (Figure 2). The position of the probe set was determined according to the international 10–20 system, with the middle channel (CH25) of the most inferior row placed at Fpz. A 3D-magnetic space digitizer (PATRIOT, Polhemus, Colchester, VT, USA) was used to validate the position of the probes (Table 2.) All probe sets were examined and adjusted to ensure consistency in the positions among the participants.

### 2.4. fNIRS Data Preprocessing

During pre-processing, the first and last 15 s of the raw time series were removed to ensure the stability of the data. The fNIRS raw data were preprocessed using the Homer2 [28]. Specifically, the raw intensity data were first converted to optical density (OD) changes. Next, the detection and correction of motion artifacts were conducted using a discrete wavelet transformation filter, and principal component analysis (PCA) with a threshold of 80% was used to remove global physiological noises [29]. Third, a bandpass filter (0.01–0.08 Hz) was applied to reduce the effect of low-frequency fluctuations (<0.01 Hz) and high-frequency neurophysiology noises (>0.08 Hz). Finally, HbO and HbR concentration changes were computed using the modified Beer–Lambert law. Because of the better sensitivity of the HbO [30], the following analysis focused on the HbO only.

### 2.5. Network Construction

The GRETNA toolbox and NIRS-KIT toolbox were used to construct the brain functional network [31,32]. The Pearson correlations between all pairs of time series were considered as the edges of the network. Then, the correlation coefficients were transformed into z-values via Fisher’s r-to-z transformation to improve the normality. The 27 × 27 z-value matrix of each participant was then converted into a binary matrix using a sparsity method. Network sparsity was defined as the ratio of the number of real edges divided by the maximum number of possible edges in a network. This method can ensure that the networks of each group have the same number of edges in each sparsity threshold. The sparsity range of 0.13–0.4 with an interval of 0.01 was used, which was determined to ensure that the resulting network had sparse network properties, without multiple connected components or isolated nodes [33,34].

### 2.6. Network Analysis

Five global metrics, including small-worldness (σ), global efficiency (*E_gloc_*), local efficiency (*E_loc_*), clustering coefficient (*C_p_*) and characteristic path length (*L_p_*), and one nodal metric, nodal efficiency (*E_nod_*), were selected to characterize the topological properties of the brain networks in the Hangzhou and Xining groups. These parameters are typical metrics used in previous studies on fNIRS resting state networks, making them comparable with other studies [35]. Finally, the area under the curve (AUC) of all network metrics, which was the integral-over-sparsity range, was computed for subsequent statistical analysis. The formulas and interpretation of these network measures can be found in the manuscript of the GRETNA toolbox and previous studies [31,36]

### 2.7. Complexity Analysis

Multiscale entropy (MSE) analysis was applied to estimate the complexity of resting-state fNIRS signals [37]. The quantification of complexity changes can detect changes in brain activity characteristics associated with the prolonged exposure to high altitude. The procedure of MSE consists of three steps. First, the original time series were coarse-gained by averaging the adjacent data points according to different timescales (1–25). Second, the sample entropy was calculated for each coarse-grained time series (pattern length, m = 2; similarity criterion, r = 0.2). Finally, the AUC-over-timescale range in each channel was calculated for subsequent statistical analysis. The formulas and interpretation of MSE are available at http://www.psynetresearch.org/tools.html (accessed on 7 November 2021).

### 2.8. Statistical Analysis

For the performance of the N-back task, the reaction times (RT) for hits (hitRT) and correct rejections (rejRT) were calculated (trial outliers with RTs shorter than 150 ms or longer than mean ± 2.5 S.D. were excluded). Then, the performance index of the non-parametric index of sensitivity A’ was calculated based on the hit rate (H) and false alarm rate (FA). The calculation formula for A’ is [38]:

H ≥ FA: 0.5 + [(H − FA)(1 + H − FA)]/[(4H(1 − FA)],

FA > H: 0.5 – [(FA − H)(1 + FA − H)]/[(4FA(1 − H)].

An altitude-by-condition mixed-model analysis of covariance (ANCOVA) was conducted for A’, hitRT and rejRT, respectively, while age and RPMs were entered as covariates. The normality distribution of the variables was evaluated with the Kolmogorov–Smirnov test. SPSS V22.0 software (IBM Corp., Armonk, NY, USA) was used for statistical analysis.

A network-based statistic (NBS) approach (GTETNA Toolbox) was used to identify the specific network components that have significantly different functional connectivity between the two groups. Specifically, the connected components were first determined by a two-sample *t*-test with a cluster-defining threshold (*p* < 0.001). Then, a permutation test (component significance: *p* < 0.05, permutations: 1000) was applied to determine the significant components between the two groups. For network properties and complexity, two-sample *t*-tests with false discovery rate (FDR) correction (*p* < 0.05) were adopted to compare the difference between the two groups. The effect of age was regressed from the statistical analysis. The BrainNet Viewer toolbox (Beijing Normal University, Beiijng, China, https://www.nitrc.org/projects/bnv/ (accessed on 25 June 2022)) was used for the visualization of the functional connectivity and network properties [39].

### 2.9. Relationship between Behavior Performance and Brain Activity

To test the association between behavior performance and the features of resting state brain activity, Pearson correlation analyses were performed in both groups, with age and RPMs as the covariance. FDR was used for multiple correction (*p* < 0.05).

## 3. Results

### 3.1. Behavior Results

The behavioral results are shown in Table 3. No significant altitude-related effects were found between the Hangzhou and Xining groups (all *p_s_* > 0.05).

### 3.2. Functional Connectivity

**Figure 3:**
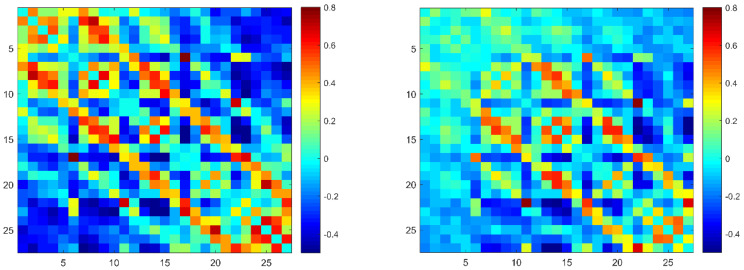
The correlation matrices of the two groups (left, Hangzhou; right, Xining; digits in the matrices represent channels).

**Figure 4:**
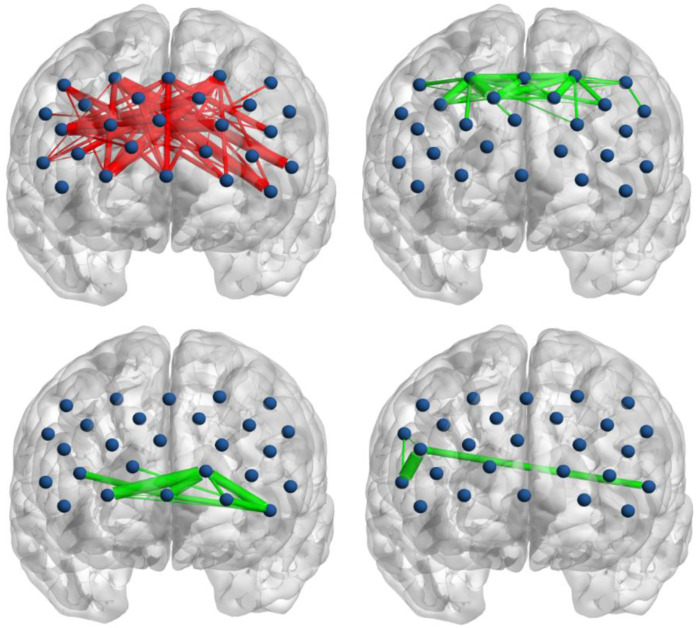
The results of functional connectivity. The red lines denote increased functional connectivity in the Xining group compared with the Hangzhou group, while green lines denote decreased functional connectivity. The thickness of the line indicates the strength of the connection. The blue dots denote 27 measurement channels.

The group-averaged functional connectivity in the two groups is shown in Figure 3. NBS analyses were used to identify the altered connected components. Compared with the Hangzhou group, one component exhibited increased connectivity, while three components exhibited decreased connectivity in the Xining group. Specifically, the component that showed enhanced connectivity was mainly located between the rostral and caudal prefrontal cortex. In contrast, two components with decreased connectivity were located within the rostral and caudal prefrontal cortex. In addition, a small component with four nodes and four connections, which was located between the right and left hemispheres, also exhibited reduced connectivity (Figure 4).

### 3.3. Global Network Properties

**Figure 5:**
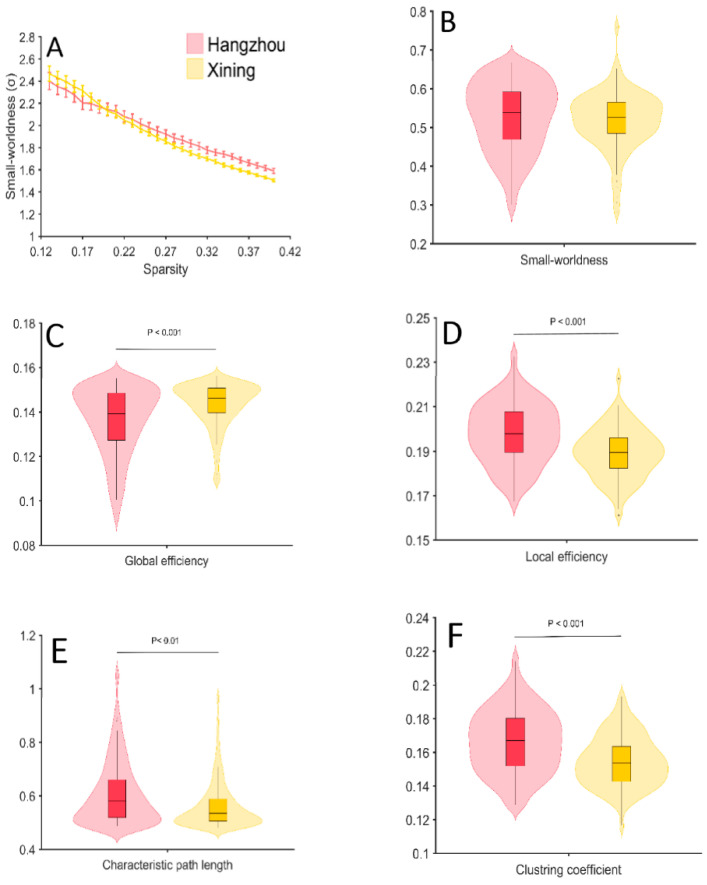
Group differences in the network properties between the two groups. (**A**) The small-worldness quantified from the Hangzhou (red) and Xining groups (yellow). (**B**–**F**) Group differences in the small-worldness, global efficiency, local efficiency, characteristic path length and clustering coefficients between Hangzhou and Xining groups.

The results of global network properties showed that, over the sparsity range, σ was greater than 1 (*p* < 0.01) in the two groups. Thus, the networks of both groups showed small-world properties. No significant difference was found in the σ between the two groups. For network efficiency, the statistical analyses indicated that the Xining group showed a significant increase (*p* < 0.001) in global efficiency and a significant decrease (*p* > 0.001) in local efficiency compared with the Hangzhou group. Furthermore, significant decreases were also found in the clustering coefficient (*p* < 0.001) and characteristic path length (*p* < 0.01) in the Xining group compared with the Hangzhou group (Figure 5).

### 3.4. Regional Nodal Properties

The results of the regional nodal properties showed that the Xining group exhibited increased nodal efficiency in most brain regions (CH3, CH6, CH7, CH10, CH12, CH13, CH14, CH15, CH16, CH17, CH18, CH19, CH20, CH21, CH25), except for CH2, which was decreased as compared with the Hangzhou group. No other significant effects were found (*p* > 0.05) (Figure 5).

### 3.5. Complexity

**Figure 6:**
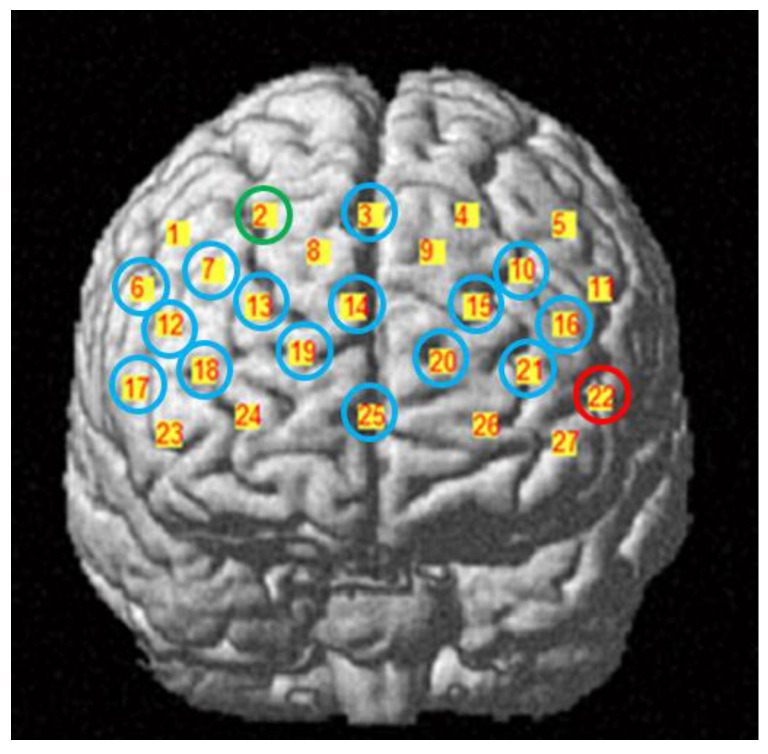
The results of nodal efficiency and complexity. The number on the yellow square background represents the position of the 27 channels. The blue circle denotes channels with increased nodal efficiency; the green circle denotes channels with decreased nodal efficiency; the red circle denotes channels with decreased complexity.

The results of the complexity analysis showed that the MSE of CH22 was significantly decreased in the Xining group compared with the Hangzhou group (*p* < 0.01). No other significant effects were found (*p* > 0.05) (Figure 6).

### 3.6. Correlation

**Figure 7:**
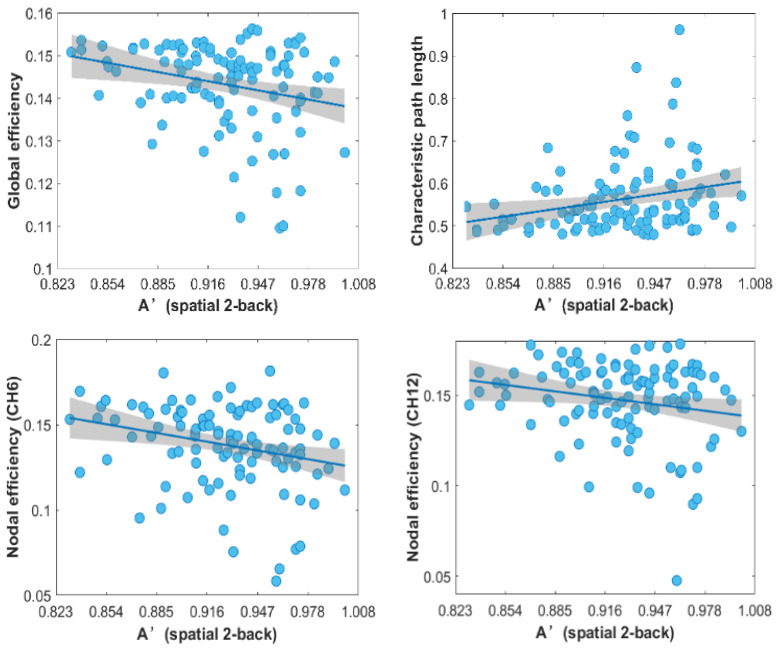

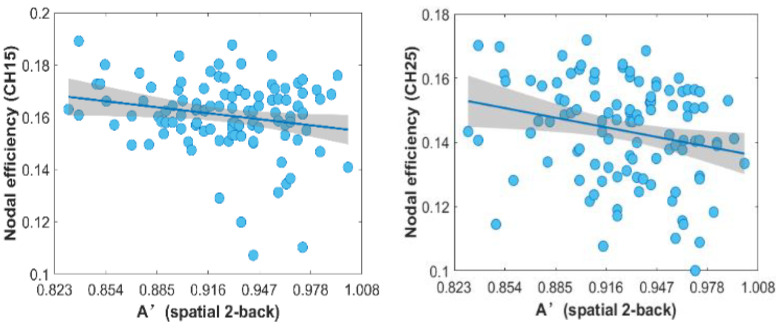
Correlation between behavioral performance (2-back A’) and network properties in the Xining group.

The results of the correlation analysis showed that the 2-back A’ was significantly negatively correlated with the global efficiency (r = −0.314, *p* < 0.01) and significantly positively correlated with the characteristic path length (r = 0.273, *p* < 0.05) in the Xining group. For nodal efficiency, four channels (CH6, CH12, CH15, CH25) showed significantly negative correlations (*p* < 0.05) with 2-back A’ in the Xining group (Figure 7). No other significant correlations were found (*p* > 0.05).

## 4. Discussion

The present study examined the effects of two years of exposure to 2260 m altitude on working memory and resting-state brain activity in a large sample. The main results showed that: (1) the spatial working memory did not significantly change compared with the Hangzhou sea level group; (2) the overall functional connectivity in the prefrontal cortex was increased, while the local short-distance connectivity within the rostral and caudal PFC was decreased; (3) the global efficiency was increased and the local efficiency was decreased in the PFC; (4) the complexity of the left dorsal lateral prefrontal cortex (lDLPFC, CH22) was decreased; (5) the global efficiency and nodal efficiency in the PFC was negatively correlated with spatial working memory performance.

Consistent with our hypothesis, no significant differences were found in spatial working memory performance between the Xining and Hangzhou groups. This finding is in line with a previous study that showed that acute exposure to 2600 m altitude (simulated normobaric hypoxia) did not impair spatial working memory [15]. Moreover, another study on long-term exposure to moderate altitude showed that two years of exposure to 2950 m altitude did not affect response inhibition [19]. Our findings provide further evidence that there is no detectable influence of long-term exposure to moderate altitude on spatial working memory performance, whereas a study on high altitude showed that long-term (three years) exposure to 3650 m altitude decreased spatial working memory performance [40]. This suggests that hypoxia stress under long-term exposure to moderate altitude is not sufficient to lead to the behavioral impairment of working memory.

The results of functional connectivity showed that the functional connectivity between the rostral and caudal prefrontal cortex were increased in the Xining group compared with the Hangzhou group. In contrast, decreased connectivity was found within the rostral and caudal prefrontal cortex in the Xining group. The frontal cortex is a functionally distinct and integrated dynamic network system [41,42]. The rostral PFC is primarily involved in the processing and integration of abstract information (i.e., schematic control), such as the evaluation and selection of cognitive strategies, while the mid-lateral PFC (i.e., caudal prefrontal in the current study) is involved in orchestrating broader network dynamics to complete various tasks (i.e., contextual control) [43]. The decreased connectivity within the rostral PFC and caudal PFC points to a reduced local information processing capacity after two years of exposure to 2260 m altitude. In contrast, increased connectivity between the rostral and caudal FPC indicates enhanced information communication between the schematic control and contextual control zones [43], perhaps reflecting an adaptive compensatory mechanism in the PFC.

Network analyses showed that the global efficiency was increased while the local efficiency was decreased in the Xining group compared to the Hangzhou group. Global efficiency reveals the efficiency of parallel information transmission in a network. The local efficiency measures the average of the efficiency of the neighborhood sub-networks of each node, reflecting the fault tolerance of a network. The decreased local efficiency may be tied to hypoxia-related brain damage and has been explained by the impaired functional segregation of the brain after two years of exposure to moderate altitude [44]. By contrast, the increased global efficiency may be associated with cerebral compensation. Furthermore, the characteristic path length and cluster coefficient were decreased in the Xining group compared with those in the Hangzhou group, which is consistent with the increased global efficiency and decreased local efficiency.

For nodal properties, the nodal efficiency was increased in multiple regions of the PFC. Nodal efficiency quantifies the extent of information communication between a given node and other nodes within a network, highlighting the importance of the node. Thus, increased nodal efficiency may indicate the higher information flow of the prefrontal cortex after two years of exposure. This is consistent with the increased global efficiency and might also reflect the compensation mechanism. The analyses of MSE showed that the complexity of lDLPFC was significantly decreased in the Xining group compared with the Hangzhou group. The lower entropy values in the lDLPFC imply that the lDPFC activity had strong consistency and regularity, supporting the comparable executive ability in moderate-altitude environments.

The correlation results showed that the spatial working memory performance was negatively correlated with the global efficiency and nodal efficiency, indicating that participants with lower spatial working memory performance tend to have higher information processing efficiency in the PFC in the Xining group. Considering that both the global efficiency and nodal efficiency were increased in the Xining group compared with those in the Hangzhou group, the negative correlation may once again imply a compensatory mechanism to cope with long-term exposure to moderate-altitude environments.

Some limitations of the present study should be noted. First, only the PFC was selected. It is possible that other brain regions that the probe set did not cover are involved in adaptive changes to long-term exposure to moderate altitude. Second, compared to cross-sectional studies, longitudinal studies are better for controlling for more confounding variables, such as individual psychological and physiological differences. Future research should use multimodal brain image techniques and longitudinal studies with large sample sizes to investigate individual differences in the adaptation to moderate altitudes.

## 5. Conclusions

In summary, for the first time, our study explored, with a large sample size and with the same set of fNIRS instruments, the compensational adaption mechanism of long-term exposure to moderate altitude by the combined use of a working memory task and resting-state brain spontaneous activity in the PFC. Our study suggests that there is no detectable influence of long-term exposure to moderate altitude on spatial working memory performance, and, more importantly, the brain may undergo compensatory changes in plasticity in the PFC, which may play an important role in the adaptation to hypoxia environments. The present study therefore provides novel insight into the effect of moderate altitudes on human cognitive function and brain activity. Moreover, this study has important implications for highland tourism, migration and athlete training.

## Figures and Tables

**Table 1:** Demographic and physiological information.

	Hangzhou (*n* = 91)	Xining (*n* = 117)	*p* (*t*/*χ*^2^)
Age	20.22 ± 0.48	20.41 ± 0.90	>0.05
Female/Male	46/45	65/52	>0.05
RPMs	60.34 ± 29.29	57.61 ± 27.23	>0.05
SpO_2_	98.68 ± 0.99	96.76 ± 1.26	<0.001
HR	80.55 ± 11.10	77.12 ± 9.43	<0.05

RPMs, Raven’s Progressive Matrices; SpO_2_, peripheral oxygen saturation; HR, heart rate.

**Table 2:** The MNI coordinates and anatomical labels corresponding to the 27 measurement channels.

Channel		MNI		Brain Regions (Percentage of Overlap)	Brodmann Area
	X	Y	Z		
1	47	27	47	Dorsolateral prefrontal cortex (0.63)	9
2	25	42	50	Includes frontal eye fields (0.79)	8
3	2	47	50	Includes frontal eye fields (0.75)	8
4	−20	43	51	Includes frontal eye fields (0.83)	8
5	−42	28	48	Includes frontal eye fields (0.61)	8
6	55	27	34	Dorsolateral prefrontal cortex (0.90)	46
7	38	45	38	Dorsolateral prefrontal cortex (0.69)	9
8	15	57	41	Dorsolateral prefrontal cortex (0.83)	9
9	−11	58	41	Dorsolateral prefrontal cortex (0.85)	9
10	−33	48	38	Dorsolateral prefrontal cortex (0.80)	9
11	−50	30	35	Dorsolateral prefrontal cortex (0.51)	46
12	48	45	27	Frontopolar area (0.72)	10
13	27	60	29	Frontopolar area (0.92)	10
14	6	65	31	Frontopolar area (0.90)	10
15	−21	62	29	Frontopolar area (0.84)	10
16	−42	48	26	Dorsolateral prefrontal cortex (0.66)	46
17	56	39	12	Dorsolateral prefrontal cortex (0.39)	46
18	41	60	16	Frontopolar area (1)	10
19	18	71	19	Frontopolar area (1)	10
20	−14	71	17	Frontopolar area (1)	10
21	−34	62	15	Frontopolar area (0.97)	10
22	−51	44	10	Dorsolateral prefrontal cortex (0.56)	46
23	48	55	1	Frontopolar area (0.88)	10
24	29	69	6	Frontopolar area (1)	10
25	3	70	6	Frontopolar area (1)	10
26	−23	71	4	Frontopolar area (1)	10
27	−42	59	−1	Frontopolar area (0.78)	10

**Table 3:** The results of spatial n-back performance.

	Group	0-Back	1-Back	2-Back
A’ (unit)	Hangzhou	0.948 ± 0.004	0.941 ± 0.004	0.929 ± 0.005
A’ (unit)	Xining	0.941 ± 0.003	0.936 ± 0.003	0.923 ± 0.004
hitRT (ms)	Hangzhou	471.48 ± 14.05	550.84 ± 16.91	749.96 ± 24.68
hitRT (ms)	Xining	460.56 ± 10.80	545.38 ± 12.99	696.81 ± 18.95
rejRT (ms)	Hangzhou	515.99 ± 14.31	612.06 ± 19.02	846.00 ± 25.80
rejRT (ms)	Xining	504.09 ± 11.00	603.47 ± 14.61	792.51 ± 19.81

## Data Availability

The datasets that support the findings of the study are available from the corresponding author upon reasonable request.

## References

[1] Tremblay J.C., Ainslie P.N. (2021). Global and country-level estimates of human population at high altitude. Proc. Natl. Acad. Sci. USA.

[2] Mujika I., Sharma A.P., Stellingwerff T. (2019). Contemporary Periodization of Altitude Training for Elite Endurance Athletes: A Narrative Review. Sports Med..

[3] Taylor L., Watkins S.L., Marshall H., Dascombe B.J., Foster J. (2016). The Impact of Different Environmental Conditions on Cognitive Function: A Focused Review. Front. Physiol..

[4] Shaw D.M., Cabre G., Gant N. (2021). Hypoxic Hypoxia and Brain Function in Military Aviation: Basic Physiology and Applied Perspectives. Front. Physiol..

[5] Virues-Ortega J., Garrido E., Javierre C., Kloezeman K.C. (2006). Human behaviour and development under high-altitude conditions. Dev. Sci..

[6] McMorris T., Hale B.J., Barwood M., Costello J., Corbett J. (2017). Effect of acute hypoxia on cognition: A systematic review and meta-regression analysis. Neurosci. Biobehav. Rev..

[7] Pun M., Guadagni V., Bettauer K.M., Drogos L.L., Aitken J., Hartmann S.E., Furian M., Muralt L., Lichtblau M., Bader P.R. (2018). Effects on Cognitive Functioning of Acute, Subacute and Repeated Exposures to High Altitude. Front. Physiol..

[8] Altbacker A., Takacs E., Barkaszi I., Kormos T., Czigler I., Balazs L. (2019). Differential impact of acute hypoxia on event related potentials: Impaired task-irrelevant, but preserved task-relevant processing and response inhibition. Physiol. Behav..

[9] Thakur L., Ray K., Anand J.P., Panjwani U. (2011). Event related potential (ERP) P300 after 6 months residence at 4115 meter. Indian J. Med. Res..

[10] Legg S., Hill S., Gilbey A., Raman A., Schlader Z., Mündel T. (2014). Effect of Mild Hypoxia on Working Memory, Complex Logical Reasoning, and Risk Judgment. Int. J. Aviat. Psychol..

[11] Thropp J.E., Buza P.W. (2019). Cumulative Cyclic Exposures to 8000-ft Pressurization Equivalence and Attention Network Responses. Aerosp. Med. Hum. Perform..

[12] Hewett K.J., Curry I.P., Rath E., Collins S.M. (2009). Subtle Cognitive Effects of Moderate Hypoxia.

[13] Bouak F., Vartanian O., Hofer K., Cheung B. (2018). Acute Mild Hypoxic Hypoxia Effects on Cognitive and Simulated Aircraft Pilot Performance. Aerosp. Med. Hum. Perform..

[14] Parker P.J., Manley A.J., Shand R., O’Hara J.R., Mellor A. (2017). Working Memory Capacity and Surgical Performance While Exposed to Mild Hypoxic Hypoxemia. Aerosp. Med. Hum. Perform..

[15] Komiyama T., Sudo M., Higaki Y., Kiyonaga A., Tanaka H., Ando S. (2015). Does moderate hypoxia alter working memory and executive function during prolonged exercise?. Physiol. Behav..

[16] Schlaepfer T.E., Bärtsch P., Fisch H.U.J.C.S. (1992). Paradoxical effects of mild hypoxia and moderate altitude on human visual perception. Clin. Sci..

[17] Petrassi F.A., Hodkinson P.D., Walters P.L., Gaydos S.J. (2012). Hypoxic Hypoxia at Moderate Altitudes: Review of the State of the Science. Aviat. Space Environ. Med..

[18] Zhang J.X., Liu H.C., Yan X.D., Weng X.C. (2011). Minimal Effects on Human Memory Following Long-Term Living at Moderate Altitude. High Alt. Med. Biol..

[19] Wei X., Ni X., Zhao S., Chi A. (2021). Influence of Exposure at Different Altitudes on the Executive Function of Plateau Soldiers-Evidence from ERPs and Neural Oscillations. Front. Physiol..

[20] Chen X., Liu J., Wang J., Xin Z., Zhang Q., Zhang W., Xi Y., Zhu Y., Li C., Li J. (2020). Altered resting-state networks may explain the executive impairment in young health immigrants into high-altitude area. Brain Imaging Behav..

[21] Zhang X., Kang T., Liu Y., Yuan F., Li M., Lin J., Zhang J.J. (2022). Resting-State Neuronal Activity and Functional Connectivity Changes in the Visual Cortex after High Altitude Exposure: A Longitudinal Study. Brain Sci..

[22] Davranche K., Casini L., Arnal P.J., Rupp T., Perrey S., Verges S. (2016). Cognitive functions and cerebral oxygenation changes during acute and prolonged hypoxic exposure. Physiol. Behav..

[23] Lawley J.S., Macdonald J.H., Oliver S.J., Mullins P.G. (2017). Unexpected reductions in regional cerebral perfusion during prolonged hypoxia. J. Physiol..

[24] Friedman N.P., Robbins T.W. (2022). The role of prefrontal cortex in cognitive control and executive function. Neuropsychopharmacology.

[25] Racz F.S., Mukli P., Nagy Z., Eke A. (2017). Increased prefrontal cortex connectivity during cognitive challenge assessed by fNIRS imaging. Biomed. Opt. Express.

[26] Wang K., Liang M., Wang L., Tian L., Zhang X., Li K., Jiang T. (2007). Altered functional connectivity in early Alzheimer’s disease: A resting-state fMRI study. Hum. Brain Mapp..

[27] Yan X., Zhang J., Gong Q., Weng X. (2011). Adaptive influence of long term high altitude residence on spatial working memory: An fMRI study. Brain Cogn..

[28] Huppert T.J., Diamond S.G., Franceschini M.A., Boas D.A. (2009). HomER: A review of time-series analysis methods for near-infrared spectroscopy of the brain. Appl. Opt..

[29] Long Y., Zheng L., Zhao H., Zhou S., Zhai Y., Lu C. (2021). Interpersonal Neural Synchronization during Interpersonal Touch Underlies Affiliative Pair Bonding between Romantic Couples. Cereb. Cortex.

[30] Hoshi Y. (2007). Functional near-infrared spectroscopy: Current status and future prospects. J. Biomed. Opt..

[31] Wang J., Wang X., Xia M., Liao X., Evans A., He Y. (2015). GRETNA: A graph theoretical network analysis toolbox for imaging connectomics. Front. Hum. Neurosci..

[32] Hou X., Zhang Z., Zhao C., Duan L., Gong Y., Li Z., Zhu C. (2021). NIRS-KIT: A MATLAB toolbox for both resting-state and task fNIRS data analysis. Neurophotonics.

[33] Achard S., Salvador R., Whitcher B., Suckling J., Bullmore E.D. (2006). A resilient, low-frequency, small-world human brain functional network with highly connected association cortical hubs. J. Neurosci..

[34] Wang J., Wang L., Zang Y., Yang H., Tang H., Gong Q., Chen Z., Zhu C., He Y. (2009). Parcellation-dependent small-world brain functional networks: A resting-state fMRI study. Hum. Brain Mapp..

[35] Cai L., Dong Q., Niu H. (2018). The development of functional network organization in early childhood and early adolescence: A resting-state fNIRS study. Dev. Cogn. Neurosci..

[36] Rubinov M., Sporns O. (2010). Complex network measures of brain connectivity: Uses and interpretations. Neuroimage.

[37] Hager B., Yang A.C., Brady R., Meda S., Clementz B., Pearlson G.D., Sweeney J.A., Tamminga C., Keshavan M. (2017). Neural complexity as a potential translational biomarker for psychosis. J. Affect. Disord..

[38] Snodgrass J.G., Corwin J. (1988). Pragmatics of measuring recognition memory: Applications to dementia and amnesia. J. Exp. Psychol. Gen..

[39] Xia M., Wang J., He Y. (2013). BrainNet Viewer: A network visualization tool for human brain connectomics. PLoS ONE.

[40] Ma H., Zhang D., Li X., Ma H., Wang N., Wang Y. (2019). Long-term exposure to high altitude attenuates verbal and spatial working memory: Evidence from an event-related potential study. Brain Behav..

[41] Badre D. (2008). Cognitive control, hierarchy, and the rostro–caudal organization of the frontal lobes. Trends Cogn. Sci..

[42] Miller E.K., Cohen J.D. (2001). An integrative theory of prefrontal cortex function. Annu. Rev. Neurosci..

[43] Badre D., Nee D.E. (2018). Frontal Cortex and the Hierarchical Control of Behavior. Trends Cogn. Sci..

[44] Latora V., Marchiori M. (2001). Efficient behavior of small-world networks. Phys. Rev. Lett..

